# Acute management of spinal fractures: What you need to know

**DOI:** 10.1097/TA.0000000000004916

**Published:** 2026-03-24

**Authors:** Michel Paul Johan Teuben, Anna Veenstra, Hans-Christoph Pape

**Affiliations:** Department of Traumatology/Spine Center (M.P.J.T., H.-C.P.), University Hospital Zürich, Zürich, Switzerland; Department of Traumatology (A.V.), University Hospital Zürich, Zürich, Switzerland

**Keywords:** Polytrauma, spine trauma, spine diagnostics, spine surgery

## Abstract

Spinal injuries occur frequently and influence the diagnosis and treatment of concurrent traumatic conditions. Understanding of spinal trauma patterns, clinical assessment and documentation, imaging options, and classification systems is essential for making multidisciplinary treatment decisions. The initial evaluation focuses on identifying neurological deficits, assessing mechanical stability, and recognizing red-flag symptoms requiring urgent intervention. Furthermore, essential concurrent nonspinal injuries, as well as multiple spinal fractures, should be ruled out. Whole-body or whole-spine CT is the cornerstone of imaging, while magnetic resonance imaging is reserved for evaluating ligamentous injury, disc pathology, and spinal cord involvement. Internationally recognized classification systems form the basis for treatment decisions. In isolated spinal trauma, early stabilization facilitates mobilization and may improve neurological outcomes, particularly when early decompression is performed in patients with spinal cord injury. In polytrauma, however, spinal surgery must be balanced against life-threatening conditions and physiological instability. Early mobilization, respiratory support, hemodynamic optimization, and thromboprophylaxis remain critical components of postoperative care. (*J Trauma Acute Care Surg*. 2026;100:850-862.

Spinal injuries can occur in young people following high-impact trauma or in older adults following trauma of varying intensity. Timely diagnosis and treatment usually require an interdisciplinary approach, particularly for polytraumatized patients. This review explores the importance of spinal fractures in the management of patients with isolated injury and polytrauma.

## RELEVANT SPINAL ANATOMY AND EPIDEMIOLOGY OF SPINAL TRAUMA

The spine consists of 33 stacked vertebrae that articulate anteriorly via intervertebral discs and posteriorly through facet joints. The spinal canal runs within osseous structures, and nerves exit the spinal canal bilaterally through the neuroforamina.^1^ The risk of neurological deficit can be due to osseous displacement of the vertebra or their junctions.

The cervical spine is divided functionally into the axial (C0-C2) and the subaxial spine (C3-C7). The segment C0/C1 is mainly responsible for flexion and extension of the head, whereas the C1/C2 segment enables head rotation.^2,3^ In a study from Norway, subaxial fractures are more frequent than axial injuries (respectively 55% vs. 38%), with 7% of patients having combined axial and subaxial fractures.^4^ Isolated bony fractures are more frequent in older patients, whereas ligamentous involvement is typical in young patients sustaining motor vehicle accidents. However, studies on aging populations show high incidences of injured elderly patients with significant comorbidities being injured due to low-impact trauma.^4,5^ Neurological impairment is more frequently observed in patients with subaxial injuries.^4^

The thoracic spine is the most rigid area of the spine due to its kyphotic form and additional anterior stabilizing effects from the chest wall. The thoraco-lumbar junction allows, due to its specific angulation of facet joints and large-sized intervertebral discs, profound force distribution and transfer while handling extensive flexion and extension bending forces as well. The thoraco-lumbar (TL) junction is defined as the area between the 11th thoracic vertebral body and the 2nd lumbar vertebral body.^6,7^

This area is an important junctional area (as the cervicothoracic junction is), as it connects rigid (thoracic) with flexible (lumbar) regions. Consequently, most fractures occur in this junctional area [70% (TL-junction) vs. 30% (lumbar and thoracic spine)].^8,9^

In addition, insufficiency fractures, related to fragile bone stock, may also lead to complaints in patients without a clear history of falls. The sacrum is frequently involved following low-impact trauma in geriatric patients. In pathologic fractures, it is important to identify underlying causes such as osteoporosis, malignancy (mainly metastasis), or infection. Therefore, in cases of atraumatic or low-energy fractures, a subsequent diagnostic workup is warranted. Sampling for histologic and microbiological analysis is helpful.^10,11^

**Figure 1:**
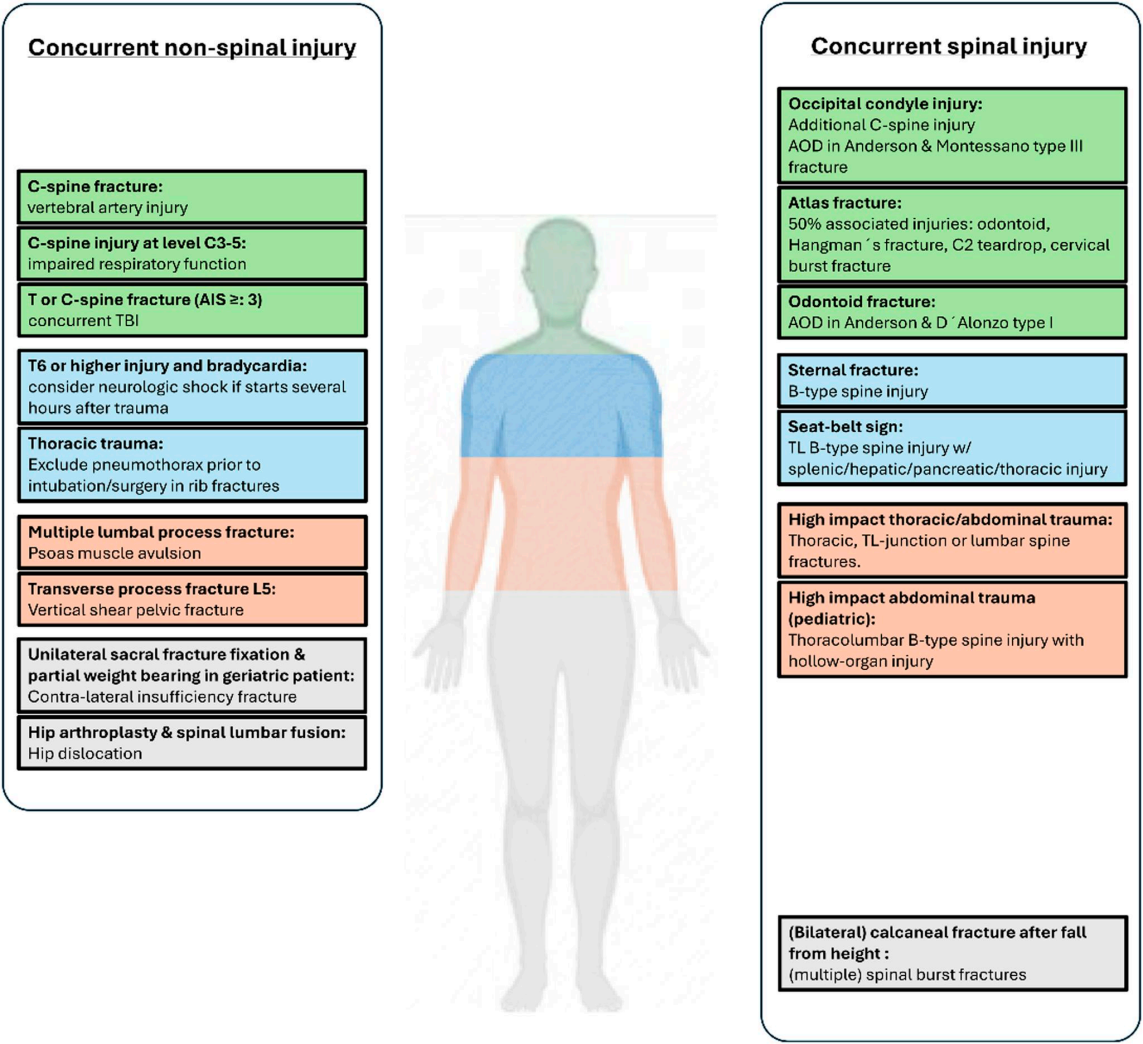
Concurrent injuries in patients with acute spinal trauma. This figure provides an overview of common combinations of injuries and relevant conditions in patients diagnosed with spinal trauma.

In direct blunt trauma, injuries mainly occur in the region with the most extensive force distribution. Any related injuries should therefore be expected to occur close to the injured vertebral body. Specific concurrent injuries should be sought out and ruled out. Patterns of concurrent injuries have been described for both thoracic and lumbar spinal injuries.^12,13^ Figure 1 provides an overview of frequent injury combinations that should be investigated, and this topic will be discussed in more detail later on.

Isolated penetrating injuries to the spine are rare. Given the high rates of more severe concurrent injuries, it is essential to rule out potential life-threatening conditions such as organ or vessel injuries before surgical spinal intervention.^14^

## CLINICAL EXAMINATION, DIAGNOSTICS, AND FRACTURE CLASSIFICATION

In parallel with emergency room investigations according to the Advanced Trauma Life Support® guidelines, a focused spinal examination should be performed. However, the ATLS guidelines consider spinal examination, including logrolling the patient, to be part of the exposure (E) assessment. It is worth noting that cervical spine protection is initiated concomitant with the airway (A) assessment, whereas a gross neurological examination is part of the disability (D) assessment.^15^

A focused spinal clinical examination should include inspection of adjacent soft tissues near the spine. The back should be checked for hematoma, open wounds, burn injuries, and scars from previous operations, as well as open fractures and potential lumbar fascial deglovement injuries (Morell-Lavallee), and for any remaining penetrating injuries or embedded objects. Furthermore, the alignment of the spine should be visually inspected; major C-type fractures can result in translation of the coronal and/or sagittal alignment of the spine. Palpation of the spinous processes should then be performed to identify local pain or dorsal gaps. The spinal examination can be performed during logrolling of the patient and may be repeated in detail during the secondary survey.^16–18^

In addition, the patient’s neurological status should be assessed, which involves defining the sensory and motor functions. In the case of cord syndromes, the level at which normal sensory and motor function is lost is reported. A rectal examination and testing the sphincter tone are also mandatory. The neurological findings should be reported in line with the International Standards for the Neurological Classification of Spinal Cord Injury (ISNCSCI), also known as the American Spinal Injury Association (ASIA) form. This allows an ASIA impairment scale to be extracted. This scale ranges from ASIA A (complete lesion with no sensory or motor function preservation in the sacral segments S4–S5) to ASIA E (normal sensation and motor function in all spinal segments).^19^

The location of the injury determines which extremities are affected. For instance, injuries to the cervical spine can lead to quadriplegia or tetraplegia, whereas injuries to the lower spine can result in paraplegia. And lesions above C6 frequently affect respiratory capacities.^20,21^ Furthermore, lesions may be complete or incomplete. Specific patterns of incomplete spinal cord injuries (SCIs) have been identified.

Central cord syndrome is the most common type of SCI in the Western world.^22^ First described by Schneider and colleagues in 1954, central cord syndrome is characterized by more severe neurological impairment of the upper extremities than the lower extremities, and, by definition, involves intact rectal tone and is an incomplete injury. In addition, bladder retention frequently occurs. The typical mechanism of injury is hyperextension trauma in pre-existing spinal stenosis, which explains its high incidence in elderly patients. Initially, this distribution was attributed to the more central localization of upper extremity fibers in the corticospinal tract of the central cord.^23^ However, more recent studies have challenged this theory, shifting the focus to the concept of diffuse white matter injury.^24^

Every physician involved in the treatment of patients with spinal injuries needs to identify the so-called red flags:

- acute loss of motor function,
- acute loss of sensory function, and
- new urine or fecal incontinence.

These red flags warrant prompt consultation with a spine surgeon or neurologist.

For severely injured trauma patients who are physiologically stable, imaging studies will be performed immediately after the primary survey. The standards for imaging trauma patients differ between institutions, with either initial conventional x-ray studies combined with ultrasound investigations or instant computed tomography imaging.^15^

Spinal fractures are seen in ~30% of polytrauma patients, with lumbar fractures being particularly prevalent.^13,25^ Before the widespread use of whole-body trauma CT scanning, up to 30% of spinal fractures were missed.^26–28^ Therefore, the use of CT imaging in polytrauma patients with suspected spinal injuries is highly recommended.^29–31^ Specific reconstructions can be considered to specifically visualize the spine.^32^

Furthermore, specific injury patterns and combinations must be identified, and patients with specific mechanisms of injury present with a predictable pattern of injuries.^12,33^

As anticipated, lumbar and thoracic spine injuries are both associated with high rates of concurrent abdominal but also chest injuries.^34^ Patients presenting with a seatbelt sign are likely to have thoraco-abdominal solid organ injuries or rib fractures.^12,13,34^ Spinal imaging should be performed in these patients as well. In cases of diagnosed sternal fractures, a high thoracic spinal lesion, frequently a B-type injury, should be ruled out.^35^

Traumatic lung injuries such as contusions and lacerations are frequent in patients with thoracic spine injuries. Therefore, thoracic trauma workup should include the assessment of the thoracic spine and the adjacent junctional zones.^13,33,36^

In general, it is recommended to rule out lumbar spine injuries in patients with pelvic fractures, abdominal organ injuries, and those after falls from a height.^37^ This mechanism of injury frequently leads to lumbar burst fractures with bilateral calcaneal fractures (known as lover´s leap).^38^

A higher number of spinal fractures is linked with higher odds of concurrent solid organ injuries. Therefore, expanding imaging to total-body CT scanning can be considered in patients with multiple spinal injuries.^37^

Especially in older patients, C-spine imaging should be considered in those individuals with expected craniocerebral injuries.^39,40^ Patients with diagnosed spinal injuries potentially involving structures surrounding the vertebral arteries require workup to rule out occlusions, dissections, and other types of vascular injuries (such as vertebral artery injuries in patients with C-spine injuries at the level C1–C3, facet dislocations, or transverse foramen involvement).^41,42^

Interestingly, a recent study on severely injured patients with spinal trauma revealed that the association of head injuries in polytrauma patients with cervical spinal injuries and thoracic spinal injuries is significant. Therefore, a CT scan of the head may be considered for all patients with either thoracic or cervical spinal injuries. A study on more than 12,000 trauma patients further demonstrated that 7.2% of patients were diagnosed with multiple spinal injuries. The most frequent fracture overseen was the second spine fracture. Consequently, whole-spine imaging is recommended for polytrauma patients with an initially diagnosed spinal fracture.^12,43^

In less severely injured patients with suspected cervical spinal trauma, the Canadian C-spine rules, NEXUS criteria, or Western Trauma Association cervical spine clearance criteria can be utilized to determine the need for additional imaging.^40,44,45^ Especially in older patients, however, low thresholds for imaging should be applied to prevent missing injuries.^46^ Besides, a specific clinical decision rule for thoraco-lumbar spinal injuries has been proposed as well.^47^

The need for emergency magnetic resonance imaging (MRI) studies is heavily debated, with differing indications among institutions.^48^ MRI studies may be used to assess ligamentous injuries, disc involvement, and narrowing of the spinal canal or neuroforamen, as well as to distinguish between old and new bony lesions (bone bruising) and dural tears/leaks. If there are neurological deficits, MRI imaging can help determine the extent of the required decompression.^48,49^ Furthermore, in spinal injuries, disc involvement dictates the need for an anterior approach in B-type fractures. If patients have unclear neurological deficits, an MRI scan can be helpful.^26,27^ In multilevel injuries with neurological abnormalities, an MRI scan may help differentiate between the impact of the injuries and the neurological impairment. MRI investigations are also indicated for patients with spinal ankylosing disorders (such as M. Bechterew, diffuse idiopathic skeletal hyperostosis, and end-stage advanced spondylosis multiplex) to identify occult fractures and multilevel injuries. Finally, if a ligamentous injury cannot be ruled out before initiating conservative management, MRI studies are indicated.^26,27,49,50^ Furthermore, MRI investigations are helpful in the workup of suspected Spinal Cord Injury Without Radiological Abnormality (SCIWORA), both in pediatric and adult patients.^51^

In general, to determine the treatment plan, it is essential to perform imaging (CT with or without MRI) to gain information on the following aspects of the fracture:

- posterior wall involvement,
- facet joint involvement, and
- tension band involvement.

Imaging studies also enable fractures to be classified, which informs later treatment decisions. Several classification systems have been developed for fractures. The AO-Spine classification is the most frequently utilized system for thoracic and lumbar fractures, and it categorizes fractures into three types. Type A fractures are compression injuries. In these fractures, the assessment of the involvement of the posterior elements of the vertebral body is essential. Type B fractures are distraction injuries implying tension band involvement, whereas type C fractures are translational or dislocated injuries. This classification helps guiding treatment decisions. Other scoring systems have been developed, such as the Thoracolumbar AO-Spine Injury Score, which integrates the morphologic AO-Spine classification, neurological status, and two specific modifiers. Specifically, these are the presence or absence of injury to the posterior ligamentous complex and patient-specific factors.^52,53^

In addition, a more specific classification system has been developed for the upper cervical spine region (the AO-Spine Upper Cervical Injury Classification System).^54^ In this classification system, type A injuries have no ligamentous involvement and are considered stable. Type B injuries have tension band or ligamentous injury and may be unstable. Type C injuries are characterized by significant translation and loss of anatomic integrity and are considered unstable.^54^

## INDICATIONS FOR SPINAL SURGERY

Once the diagnosis of all spinal injuries is determined and all other concurrent injuries have been identified and classified, a management plan is devised. To determine the need for spinal surgery, the following injury aspects must be assessed:

- neurological deficit,
- spinal stability, and
- spinal malalignment or dislocation.^55–59^

The subsequent aims of spinal interventions are

- to adequately decompress the spinal canal and/or nerve roots;
- to stabilize injured segments while retaining mobility in the unaffected segments; and
- to reduce the dislocated elements and restore spinal alignment.

Neurological impairment (i) following spinal injury can be determined using the previously described neurological examination and ASIA scoring, as well as spinal imaging, including CT and MRI studies. It is also necessary to rule out other causes of neurological impairment, such as concurrent traumatic brain injury (TBI) or intoxication.^26,48,49,60^

A key criterion mandating surgical stabilization is the degree of fracture stability (ii), which is defined as the ability to maintain spontaneous vertebral alignment. As mentioned before, the AO classification of vertebral fractures incorporates osseous destruction of the vertebral body and dorsal elements, as well as ligamentous injuries, thereby enabling a complete assessment of fracture stability.^52–54^ In borderline situations, dynamic adjuncts are needed to identify instability. These include dynamic imaging studies, follow-up imaging studies after mobilization, and alterations in pain scores over time, both with and without bracing/mobilization.^27^

Generally, worsening spinal alignment, loss of vertebral body height, persistent or increasing pain over time, or instability during dynamic imaging studies suggest instability and may necessitate surgical stabilization.

Parameters used to determine spinal misalignment or dislocation (iii) are extracted from imaging studies. C-type fractures are characterized by translation in any plane and therefore require reduction and fixation. For non–type C fractures, the degree of sagittal plane deformity should be calculated. The Cobb angle can be used to assess the impact of a spinal fracture on the alignment of the affected and adjacent spinal levels. Excessive and progressive kyphosis may require surgical intervention. Inability to preserve or restore spinal alignment will lead to spinal issues later on in life and should be avoided.^58,61^

Contraindications for (spine) surgery include cardiopulmonary instability, persistent hypothermia, coagulopathy, and metabolic imbalances, including acidosis, and the need to temporarily stabilize long-bone fractures first or severe TBI.^62,63^

The priorities of trauma care and the timing of surgery in patients with and without neurological deficits will be discussed later.

## IMPACT OF SPINAL FRACTURES ON THE DIAGNOSIS AND TREATMENT OF CONCURRENT INJURIES

Patients with trauma and accompanying intra-abdominal solid organ injuries may undergo early spinal fracture fixation.^64^ Spinal injuries may affect both the diagnosis, treatment, and outcome of concurrent injuries and vice versa.^65–69^ This is especially the case if spinal surgery is mostly performed in the prone position.^70^ Unfortunately, there is a lack of evidence on the interplay between the treatment of spinal and nonspinal injuries. Nevertheless, Markert and colleagues have demonstrated that the failure rate of nonoperative management of solid abdominal organ injuries is not increased by early surgery in the prone position. However, they advise caution in patients with high-grade splenic injuries.^71^ Patients operated on the Jackson spine table had experienced the least cardiovascular changes compared with alternative ways of prone surgery.^67^

SCIs can impact the overall outcome of patients with multiple injuries in several ways. This is especially the case when altered pain perception masks potential injuries or clinical deterioration. For example, spinal injuries with SCI above the T6 level make it more difficult to perform a clinical examination of the abdomen, increasing the risk of missing signs of peritonitis or a complicated clinical course during trials of nonoperative management, and this risk persists lifelong.^72,73^

Furthermore, as SCIs require increased systemic blood pressure levels, there is an increased risk of bleeding complications from solid organ injuries.^74,75^

Higher spinal cord or nerve root injuries may further affect respiratory function. The nerve roots of C3, C4, and C5 are responsible for maintaining diaphragm movement during the respiratory cycle. Diaphragmatic insufficiency due to neurological injury may necessitate lifelong ventilatory support and increase the risk of respiratory complications during the early posttraumatic period.^20^

In patients with concurrent TBI, the impact of prolonged prone positioning on intracranial pressure should also be considered.^76^ An interdisciplinary risk-benefit analysis should be conducted. Individuals with severe TBI can only be cleared for spinal fracture fixation if intracerebral bleeding (based on imaging) and intracranial pressure (levels below 15–20 mm Hg) have stabilized.^62,63^ If it is unclear whether patients can tolerate prone positioning for spinal interventions, a trial can be performed in the ICU before clearing the patient for spine surgery.

The utilization of a halo vest, as an alternative for surgical stabilization for cervical fractures, is a topic of high debate. Specifically, in the elderly, this is associated with impaired outcome.^77^

## TIMING OF SPINAL SURGERY IN ISOLATED SPINAL TRAUMA

When spinal surgery is indicated, prompt spinal stabilization improves early pain relief and facilitates early mobilization.^62,64,78,79^ Interestingly, the beneficial effect on outcome (hospital and ICU stay and lower complication rates) is more profound in patients with higher injury severity scores.^80^ In cases of SCI, the concept of “time is spine” applies. Early surgical decompression within 24 hours of an acute SCI has been linked to improved outcomes.^79,81^ The first 24 to 36 hours following injury are considered a crucial period during which decompressive surgery can significantly improve neurological outcomes.^81–83^

Other factors affecting outcome after SCI include the presence or absence of hemorrhagic shock. The management of hemodynamic instability can particularly influence the degree of neurological damage due to secondary ischemia. Another important factor is the neurological level of injury, as it determines the amount of space available for the spinal cord to expand without decompression surgery or spontaneous decompression through fracture of the dorsal elements of the spine.^84,85^

## TIMING AND PRIORITIZING (SPINAL) SURGERY IN POLYTRAUMA PATIENTS

In polytrauma patients, creating a treatment plan that determines the priority and order of interventions is essential. To prioritize interventions adequately, it is important to consider the injuries and the patient’s physiology. Patients should be cleared for each intervention individually, and sometimes it is preferable to postpone surgery to first optimize the patient’s physiology in the intensive care unit. The physiological factors that should be monitored are based on the “lethal triad.”^86^ More precisely, hypothermia, acidosis, and coagulopathy are conditions that should be aggressively managed in the initial phase after trauma. In addition, systemic inflammatory complications such as acute respiratory distress syndrome and multiple organ dysfunction syndrome should be prevented. Therefore, in line with early appropriate care and safe definitive care protocols, surgery or interventions that could lead to significant blood loss should be postponed.^87–89^

In polytrauma patients with neurological deficits due to spinal trauma, systemic outcome variables (eg, early mortality due to hemorrhage, infection, multiple organ failure, and deterioration of TBI) and spine outcome variables (eg, worsening of neurological status) should be considered when clearing patients for spinal surgery.

The literature suggests that the performance of early major spine surgery (early total care) may be associated with impaired outcomes in polytrauma patients. However, stable polytrauma patients may benefit from early stabilization (<72 h).^62,64,78,90^ McLain and Benson demonstrated in a prospective study of polytrauma patients that prompt spinal stabilization is safe in selected patients. This is particularly true when procedures requiring a posterior approach are performed, as these are associated with less blood loss than anterior approach procedures.^91^ Indications for early spinal stabilization included neurological deficit, fracture instability, or severe deformity that compromised the patient’s skin or vital functions.^62,64,78,90–92^ In addition, a systematic review by Dimar et al.^80^ implies that early spinal fracture fixation is associated with better outcomes, particularly in more severely injured patients. This applies not only to patients with neurological deficits but also to those without SCIs.^62,64,78,80,90,92^ A subsequent review suggests that, according to the damage control concept, early posterior fixation of unstable fractures is preferable to a more invasive 360-degree intervention.^64^ However, controversy still exists regarding the benefits of early intervention in specific spinal regions, such as the cervical spine.^12,93^ Further research should focus on this issue. In contrast, the benefits of more aggressive early treatment in polytrauma patients with thoraco-lumbar fractures with and without SCIs are well-established, as it is associated with fewer complications likely due to better mobilization and less secondary neurological deterioration in individuals with SCI.^62,64,78,80,90–92,94^ A multidisciplinary expert panel recommended surgical intervention within 24 hours.^75^ Severe thoracic trauma and low hemoglobin levels are associated with an impaired outcome following early stabilization.^95^

Based on the current literature, it seems feasible to achieve spinal stabilization as early as possible, especially in patients with SCIs. However, it should be delayed until the polytraumatized patient is stable.^62,64,78,80,90–92,94^ If decompression is not required, a two-stage approach, involving minimal invasive interventions with percutaneous techniques, as part of damage control principles, should be considered, especially in fragile patients.^96–98^ Of note, percutaneous techniques can also be combined with in-between selective decompression.

So, when integrating spinal fractures into the basic concepts of prioritizing trauma care and international guidelines, life-threatening surgery should be performed first. This rarely involves spinal injuries, except for atlanto-occipital injuries/dislocations, high cervical spine injuries, and near-vascular spinal trauma. Thereafter, the concept of “safe life, safe limb” applies. If the viability of the limbs is not at risk, it is recommended to start with the fixation of proximal long-bone injuries, followed by distal ones, as this optimizes mobilization and prevents the development of venous thromboembolic events (VTEs). Other fractures and ligamentous injuries should then be addressed. It should be noted that open fractures and dislocations affect the prioritization further.

**Figure 2:**
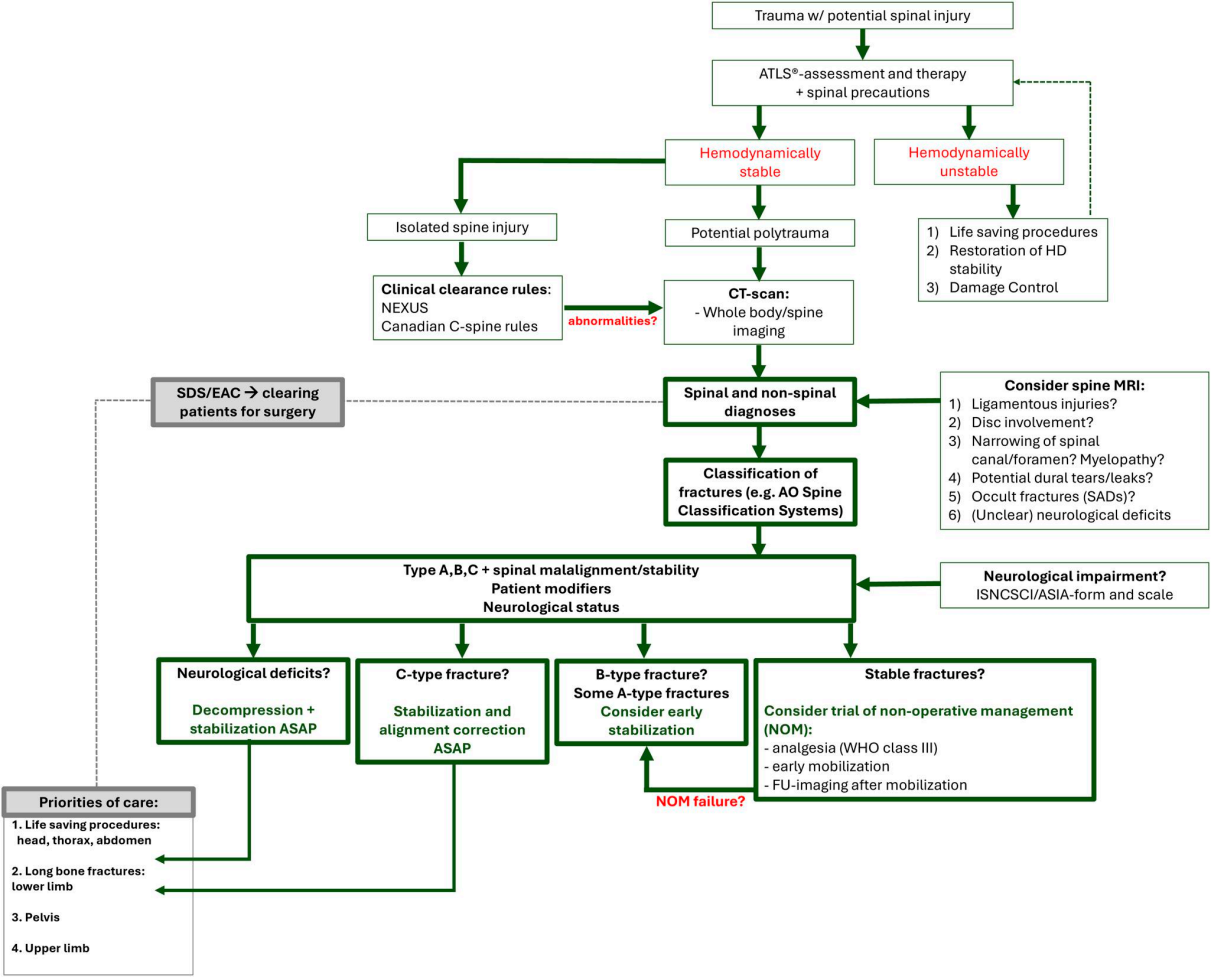
Management of acute spinal injuries. This flowchart provides an overview of diagnostics, relevant classification systems, and treatment (indications, priorities, and timing) for patients with acute spinal trauma.

In cases of SCI with neurological impairment, especially incomplete SCI, the order of surgery may also shift, as a nonfunctioning leg is worse than a broken leg. Figure 2 shows an overview of the diagnostics, classification, and treatment options for adult spinal trauma cases.

## SPINAL TRAUMA IN PEDIATRIC PATIENTS

Pediatric spinal trauma is rare but is associated with high morbidity and mortality. Diagnostics are difficult due to factors such as anamnesis and physical examination, as well as radiation exposure related to x-ray and CT imaging. Treatment is also challenging due to the factor of future growth.^99–101^

Due to its biomechanical characteristics, the pediatric spine is vulnerable, with cervical spine injuries occurring twice as frequently as in adults. Spinal cord involvement is also relatively frequent in pediatric patients. Concurrent gastrointestinal injuries are associated with thoraco-lumbar or lumbo-sacral spinal injuries, especially Chance fractures.^102,103^ SCIWORA and apophyseal injuries are conditions primarily observed in children.^104,105^

SCIWORA is the result of the higher flexibility of the pediatric spine and is linked to local ischemia or contusion in patients under 8 years of age.^105^ SCIWORA patients may present with high variability in neurological symptoms and sometimes delayed neurological deficits.^104^ There is no evidence to support the routine use of high-dose intravenous steroids in SCIWORA, and treatment mainly involves restricting physical activity for up to 6 months.^104,106,107^ MRI is often recommended as it helps to prevent unnecessary imaging with radiation exposure, distinguish between fresh injuries and ossification lines, and diagnose SCIWORA in individuals with neurological impairment.^106,108–110^

Ossification lines are often mistaken for fractures.^108,111^ Unlike fractures, ossification lines are clean and symmetrical, and are characterized by subchondral sclerotic lines. They are also seen at specific locations and disappear over time. By the age of 12, ossification centers in the pediatric cervical spine should be fused.^111,112^ Interestingly, noncontiguous spine fractures often occur in children and must be ruled out. Apophyseal injuries mainly occur in adolescent males at the L4-L5 level. If this condition results in anterior dislocation and subsequent compression of the spinal canal, surgical removal is the preferred treatment.^113^

The Pediatric Emergency Care Applied Research Network (PECARN) prediction rule is suggested to determine the need for additional imaging in pediatric patients with potential spinal injury.^101,114^

The treatment of spinal injuries in pediatric patients is similar to that for adults, as are the debates surrounding the use of methylprednisolone. Most low-grade injuries can be treated nonsurgically (including HALO or bracing), but stabilization or fusion may be required in more severe cases.^115,116^ Short constructs and fusionless surgery are preferable, with early implant removal leading to preservation of disc viability. The goal is to optimize the spine’s and associated body regions’ (eg, the thoracic cavity and lungs) later growth potential and restore alignment.^117,118^ In general, indications for surgical intervention include neurological impairment with spinal cord compression, as well as unstable fractures and increasing deformity. Wiring techniques and sublaminar hooks are useful adjuncts to screw fixation in pediatric patients.^119^ As with adult spinal trauma, no consensus has been reached on the timing of surgery, although early surgery seems to be beneficial, especially in the case of SCI.^120,121^

It is of utmost importance to further realize that spinal injuries may be related to child abuse, and this should be considered in all cases, particularly if there are multiple injuries or bruises on the proximal parts of the extremities or trunk.^122^

## POSTOPERATIVE CARE: EARLY MOBILIZATION AND REHABILITATION

Early fracture stabilization is essential as it allows for early mobilization of patients. Early mobilization correlates with improved outcomes.^123^ This is important regardless of the level of injury. Patients with no neurological deficits in the cervical spine are usually mobilized on postoperative day 1 and can leave the hospital soon afterwards. In contrast, patients with hemiplegia or worse, tetraplegia, are at high risk of pneumonia, so early fixation should be followed by early mobilization to reduce the duration of the recumbent position.^123,124^

Early mobilization can also be achieved in patients with multiple injuries, and is particularly important in the ICU setting. There, it is crucial to enable the patient to turn or sit, to improve hygiene and pulmonary toilet. A physiotherapist should be available daily in the ICU to support adequate mobilization and respiratory muscle training. Adequate painkilling should help patients to achieve adequate and pain-free sleeping, and to sit, stand, and later walk as soon as possible.^125–127^

In patients with cervical injuries, bedside dysphagia screens should be performed routinely to prevent delayed diagnosis and to prevent dysphagia-related complications.^128^

## SPECIAL CONSIDERATIONS

### Permissive Hypotension

Permissive hypotension is a resuscitation strategy involving restricted fluid administration in trauma patients. It aims to reduce complications by accepting less than physiological levels of systolic blood pressure (ranging between 80 and 90 mm Hg) and can be considered in major spinal surgery.^129^ However, it is currently unclear whether patients with spinal injuries benefit from restricted volume therapy.^130,131^ This is especially pertinent given that these protocols conflict with treatment guidelines for SCI, which recommend maintaining mean arterial pressure goals above 85 mm Hg for several days.^74,132^

### Use of Steroids in SCI

Discussions about the value of steroid use in cases of spinal injury with acute neurological deficit date back more than two decades, and the issue has been the subject of controversy. In 2002, the AANS/CNS published guidelines stated that there was insufficient evidence to recommend the use of high-dose methylprednison (MPSS) as standard therapy for SCI.^133^ The updated guidelines in 2013, however, recommended against the use of MPSS for the treatment of those with acute SCI.^134^ These recommendations are associated with a profound drop of utilization of MPSS in the United States.^135^ The 2017 AO-Spine guideline aimed to reconcile the differences between the 2002 and 2013 AANS/CNS guidelines and to recommend a 24-hour infusion of high-dose MPSS for adult patients presenting within 8 hours of acute tSCI.^136^ Further prospective studies identifying subsets of patients benefitting most from MPSS are needed.

### Patient Monitoring and Neuroprotective Targets in the ICU and OR

Hemodynamic optimization is generally recommended to improve local blood flow to the spinal cord. Current resuscitation guidelines should address the consequences of general blood loss and spinal shock. Therefore, invasive blood pressure monitoring is recommended upon admission, along with maintaining a mean arterial pressure between 85 and 90 mm Hg for several days. Special consideration should be given to preventing hypoxia and acidosis.^74,75^

Furthermore, hemoglobin levels below 7 g/dL should be corrected by transfusion, and platelet counts should exceed 50,000/mm^3^; higher levels of over 75,000 should be aimed for during spinal surgery. Hypoglycemia and PaO_2_ levels below 60 mm Hg and PaCO_2_ above 40 should also be avoided.^75^

Internationally accepted standards for trauma resuscitation should be incorporated in the treatment protocols as well. This may include the use of a REBOA resuscitative endovascular balloon occlusion of the aorta, especially in hemodynamically unstable patients or before reduction of severely displaced spine fractures with the risk of aortic injury during reduction.^137–139^

### Indications and Timing of a Tracheostomy in Patients With Spinal Injuries

In general, the need for a tracheostomy depends on whether pulmonary insufficiency has occurred or is likely to occur in the future. This may be due to an underlying disease (ie, independent of the injury), associated pulmonary contusions, biomechanical instability (eg, flail chest), and spinal cord or nerve root injury. A combination of these causes is also frequently seen. Eventually, a mismatch between respiratory demands and reserves constitutes the final indication for the procedure. In patients with a cervical spine injury or a high-level neurological deficit, subsequent muscular weakness affects respiratory capacity. As these patients usually undergo surgical fixation of the cervical spine, the timing of tracheostomy and, more specifically, combined surgery should be evaluated.^21,96,140^

As the approaches for tracheostomy and anterior cervical spine intervention are close to each other but affect different soft tissue layers, combined surgery is possible. This is technically the best option in terms of the risk of infection at the fracture site. Provided the necessary surgical expertise is available, it is feasible to perform C-spine surgery first, close the soft tissues, and perform the tracheostomy immediately afterward in a single procedure.

However, if surgical fixation has initially been performed on an emergent basis and the need for a tracheostomy is recognized subsequently, this intervention can be performed at any time. It is of great importance to prevent contamination of the other surgical wounds.^141,142^

If a tracheostomy has been performed initially, fixing an underlying fracture is technically more challenging, so all measures must be taken to minimize the risk of infection. When draping the patient, care must be taken to separate the tracheostomy tube and its skin fixation from the surgical field for sterility reasons. To ensure a clean environment, it may be necessary to replace the tube before draping. Special care must then be taken to avoid displacing the tube throughout the procedure. In addition, soft tissue dissection requires greater caution to ensure that the dissection of tissue planes required for spine exposure and fixation does not “invade” the tissue layers in the proximity of the tracheostomy. Earlier tracheostomy (before day 7) is associated with a superior outcome compared with later surgery.^96^ The trach score can be utilized to predict the need for a tracheostomy.^140^

### Posttraumatic VTE Prophylaxis in Spine Injuries

Patients with trauma, especially immobilized patients, are prone to developing VTEs.^143^ The immobilized patient with an unstable spinal fracture is the ideal setup for thromboembolic and pulmonary complications. Therefore, surgery should be performed as early as possible. The timing of initiating VTE prophylaxis and the preferred type of anticoagulant following spinal trauma are topics of debate. Zeeshan and colleagues have demonstrated that in patients who have undergone surgery for spinal injuries with an AIS-spine score >2, starting VTE prophylaxis within 48 hours is associated with lower rates of DVT and no increased risk of bleeding or mortality. It should be noted that patients with concurrent injuries with an AIS score >2 were excluded from this analysis.^144^ A recent meta-analysis of the optimal timing of VTE prophylaxis has reinforced these findings by pooling data from 4,345 patients.^145^ In a comparable cohort of surgically treated patients with spinal injuries, a retrospective analysis of 810 patients demonstrated that thromboprophylaxis with direct oral anticoagulants was associated with lower rates of thromboembolic events than low-molecular-weight heparin (LMWH).^146^ According to Sharpe et al.,^147^ providing VTE prophylaxis before spinal surgery in 705 trauma patients further decreased the risk of pulmonary embolism without altering the risk of bleeding.

In patients with spinal trauma who were not operated on, the superiority of Xa-inhibitors over LMWH therapy in preventing DVTs has also been suggested.^148^ However, the PREVENT CLOT trial showed that VTE rates did not differ between prophylactic protocols involving either LMWH or aspirin, regardless of VTE risk profile.^149^ However, trials and prospective validation studies are currently lacking.

In the case of concurrent SCI, both systemic blood loss and local hematoma formation must be considered. However, a study by Lui et al.^150^ demonstrated that administering LMWH within 24 hours after injury is not associated with high rates of spine surgery-related bleeding (2.4%). The timing and type of DVT prophylaxis for severely injured patients with spinal trauma have not yet been investigated in detail. However, as an increasing number of injuries is associated with higher DVT rates, it may be beneficial to start DVT prophylaxis promptly rather than late, if the bleeding risk related to concurrent injuries is acceptable.

Patients diagnosed with SCIs, especially at the cervical or thoracic levels, are significantly more likely to develop VTEs than those without SCI. The highest risk of developing thromboembolic events is within the first 3 months after injury.^151^ The VTE risk in trauma patients tends to equal that of the general population after 12 to 15 months. Therefore, providing prolonged VTE prophylaxis to patients with SCI is advisable.^152^

If pharmacologic prophylaxis is not used, prophylaxis with the use of mechanical compression devices should be provided. For individuals at high risk of VTE, a combination of pharmacologic and mechanical prophylaxis should be considered.^153^

## CONCLUSIONS

The management of spinal injuries in the emergency setting requires a structured and multidisciplinary approach to determine the optimal treatment strategy. Treatment decisions are based on the extent of neurological impairment, fracture stability, and retention of spinal alignment. In cases of isolated spinal trauma, early intervention is preferred, whether or not neurological deficits are present. However, it is of the utmost importance to rule out relevant concurrent injuries before the start of spinal intervention. To achieve this, a thorough diagnostic assessment is required, with active screening for frequent concurrent injuries and multilevel spinal injuries. This often necessitates total-body or whole-spine CT imaging in more severely injured patients. To determine involvement of ligamentous structures, intravertebral disc protrusions, spinal hematomas, myelomalacia, and stenosis of the spinal cord or neuroforamina, MRI imaging may be considered.

In polytrauma patients, treatment of life-threatening injuries should be prioritized over prompt spinal surgery. Delayed fracture treatment, even for unstable fractures, may be the preferred option. However, surgical decompression in patients with neurological deficits should be prioritized over the treatment of non–life-threatening concurrent injuries in cardiopulmonary-compensated patients. In cardiopulmonary unstable trauma patients, demanding spinal surgery may be postponed, even in cases of SCI. Clearing patients for spinal fracture care depends on optimizing outcome-determining factors, including coagulation, hypothermia, and metabolic and cardiopulmonary disturbances. The use of minimally invasive (percutaneous) techniques to stabilize spinal fractures is consistent with the principles of damage control surgery.

## Supplementary Material
