# Supplementary material for: Acute management of spinal fractures: What you need to know
Source: J Trauma Acute Care Surg. 2026 Mar 24;100(6):850–62. doi: 10.1097/TA.0000000000004916 (PMC13200875; doi:10.1097/TA.0000000000004916)
Supplement: Supplementary file 1 [file ta-100-850-s001.pdf]

Journal of
Trauma and
Acute Care Surgery

CONFLICT OF INTEREST DISCLOSURE FORM

Based on ICMJE Form

**Date:**	11/19/2025
**Your Name:**	Michel Paul Johan Teuben
**Manuscript Title:**	Acute Management of Spinal Fractures: What You Need to Know
**Manuscript Number (if known):**	Click or tap here to enter text.

In the interest of transparency, we ask you to disclose all relationships/activities/interests listed below that are related or unrelated to the content of your manuscript. Disclosure represents a commitment to transparency and does not necessarily indicate a bias. If you are in doubt about whether to list a relationship/activity/interest, it is preferable that you do so.

Participants of an accredited activity must disclose all personal **financial** and **non-financial relationships**, over the previous 36 months with an **ineligible company** (formerly defined as a commercial interest). **Financial relationships** are those relationships in which the individual benefits by receiving a salary, royalty, consulting fee, honoraria, ownership interest (e.g., stocks, stock options or other ownership interest), or other financial benefits, and may affect activity content relevant to products or services of an **ineligible company**, defined as an entity whose primary business is producing, marketing, selling, re-selling, or distributing healthcare products used by or on patients.

Research grants from **ineligible companies** are financial relationships that should be disclosed, even if the funds go to the researcher's institution and not to the individual researcher.

The author's relationships/activities/interests should be defined broadly and not only related to the manuscript in question. For example, if your manuscript pertains to the epidemiology of shock, you should declare all relationships with manufacturers of treatments used in shock, even if that form of treatment is not mentioned in the manuscript.

According to federal regulations approved by the US Senate, any amount equal to above $10 USD must be disclosed. Although disclosure of the total amount is not required on this form. Authors are encouraged to search the CMS Open Payments Database found at https://openpaymentsdata.cms.gov and report on the JTACS Conflict of Interest Disclosure form ALL COI, and any other conflicts related or unrelated to the manuscript being submitted to the Journal for the last 36 months/3 years.

In item #1 below, report all support for the work reported in this manuscript without time limit. For all other items, the time frame for disclosure is the past 36 months.

If the article is accepted, all author JTACS COI forms will be published as supplemental material with the article.

		**Name all entities with whom you have this relationship or indicate none (add rows as needed)**	**Specifications/Comments (e.g., if payments were made to you or to your institution)**
		Time frame: Since the initial planning of the work	
1	All support for the present manuscript (e.g., funding, provision of study materials,	☒ None	
			Click the tab key to add additional rows.

11-19-25

		Name all entities with whom you have this relationship or indicate none (add rows as needed)	Specifications/Comments (e.g., if payments were made to you or to your institution)
	medical writing, article processing charges, etc.) No time limit for this item.		
**Time frame: past 36 months**			
2	Grants or contracts from any entity (if not indicated in item #1 above).	☒ None	
3	Royalties or licenses	☒ None	
4	Consulting fees	☒ None	
5	Payment or honoraria for lectures, presentations, speakers bureaus, manuscript writing or educational events	☒ None	
6	Payment for expert testimony	☒ None	
7	Support for attending meetings and/or travel	☒ None	

		Name all entities with whom you have this relationship or indicate none (add rows as needed)	Specifications/Comments (e.g., if payments were made to you or to your institution)
8	Patents planned, issued or pending	☒ None	
9	Participation on a Data Safety Monitoring Board or Advisory Board	☒ None	
10	Leadership or fiduciary role in other board, society, committee or advocacy group, paid or unpaid	☒ None	
11	Stock or stock options	☒ None	
12	Receipt of equipment, materials, drugs, medical writing, gifts or other services	☒ None	
13	Other financial or non-financial interests	☒ None	
14	Family Disclosure. Disclose any financial associations involving a spouse, partner, or children	☒ None	

Please place an "X" next to the following statement to indicate your agreement:

☒ I certify that I have answered every question and have not altered the wording of any of the questions on this form.

11.14.21

The Journal of
Trauma and
Acute Care Surgery

CONFLICT OF INTEREST DISCLOSURE FORM

Based on ICMJE Form

**Date:**	11/19/2025
**Your Name:**	Anna Catharina Veenstra
**Manuscript Title:**	Acute Management of Spinal Fractures: What You Need to Know
**Manuscript Number (if known):**	Click or tap here to enter text.

In the interest of transparency, we ask you to disclose all relationships/activities/interests listed below that are related or unrelated to the content of your manuscript. Disclosure represents a commitment to transparency and does not necessarily indicate a bias. If you are in doubt about whether to list a relationship/activity/interest, it is preferable that you do so.

Participants of an accredited activity must disclose all personal **financial** and **non-financial relationships**, over the previous 36 months with an **ineligible company** (formerly defined as a commercial interest). **Financial relationships** are those relationships in which the individual benefits by receiving a salary, royalty, consulting fee, honoraria, ownership interest (e.g., stocks, stock options or other ownership interest), or other financial benefits, and may affect activity content relevant to products or services of an **ineligible company**, defined as an entity whose primary business is producing, marketing, selling, re-selling, or distributing healthcare products used by or on patients.

Research grants from **ineligible companies** are financial relationships that should be disclosed, even if the funds go to the researcher's institution and not to the individual researcher.

The author's relationships/activities/interests should be defined broadly and not only related to the manuscript in question. For example, if your manuscript pertains to the epidemiology of shock, you should declare all relationships with manufacturers of treatments used in shock, even if that form of treatment is not mentioned in the manuscript.

According to federal regulations approved by the US Senate, any amount equal to above $10 USD must be disclosed. Although disclosure of the total amount is not required on this form. Authors are encouraged to search the CMS Open Payments Database found at https://openpaymentsdata.cms.gov and report on the JTACS Conflict of Interest Disclosure Form ALL COI, and any other conflicts related or unrelated to the manuscript being submitted to the Journal for the last 36 months/3 years.

In item #1 below, report all support for the work reported in this manuscript without time limit. For all other items, the time frame for disclosure is the past 36 months.

If the article is accepted, all author JTACS COI forms will be published as supplemental material with the article.

		Name all entities with whom you have this relationship or indicate none (add rows as needed)	Specifications/Comments (e.g., if payments were made to you or to your institution)
		Time frame: Since the initial planning of the work	
1	All support for the present manuscript (e.g., funding, provision of study materials,	☒ None	

		Name all entities with whom you have this relationship or indicate none (add rows as needed)	Specifications/Comments (e.g., if payments were made to you or to your institution)
	medical writing, article processing charges, etc.) No time limit for this item.		
		Time frame: past 36 months	
2	Grants or contracts from any entity (if not indicated in item #1 above).	☒ None	
3	Royalties or licenses	☒ None	
4	Consulting fees	☒ None	
5	Payment or honoraria for lectures, presentations, speakers bureaus, manuscript writing or educational events	☒ None	
6	Payment for expert testimony	☒ None	
7	Support for attending meetings and/or travel	☒ None	

		Name all entities with whom you have this relationship or indicate none (add rows as needed)	Specifications/Comments (e.g., if payments were made to you or to your institution)
8	Patents planned, issued or pending	☒ None	
9	Participation on a Data Safety Monitoring Board or Advisory Board	☒ None	
10	Leadership or fiduciary role in other board, society, committee or advocacy group, paid or unpaid	☒ None	
11	Stock or stock options	☒ None	
12	Receipt of equipment, materials, drugs, medical writing, gifts or other services	☒ None	
13	Other financial or non-financial interests	☒ None	
14	Family Disclosure Disclose any financial associations involving a spouse, partner, or children	☒ None	

Please place an "X" next to the following statement to indicate your agreement:

A. Ve[illegible]

☒ I certify that I have answered every question and have not altered the wording of any of the questions on this form.

The Journal of Trauma and Acute Care Surgery

CONFLICT OF INTEREST DISCLOSURE FORM

Based on ICMJE Form

Date: 11/19/2025

Your Name: Hans-Christoph Pape

Manuscript Title: Acute Management of Spinal Fractures: What You Need to Know

Manuscript Number (if known): Click or tap here to enter text.

In the interest of transparency, we ask you to disclose all relationships/activities/interests listed below that are related or unrelated to the content of your manuscript. Disclosure represents a commitment to transparency and does not necessarily indicate a bias. If you are in doubt about whether to list a relationship/activity/interest, it is preferable that you do so.

Participants of an accredited activity must disclose all personal **financial** and **non-financial relationships**, over the previous 36 months with an **ineligible company** (formerly defined as a commercial interest). **Financial relationships** are those relationships in which the individual benefits by receiving a salary, royalty, consulting fee, honoraria, ownership interest (e.g., stocks, stock options or other ownership interest), or other financial benefits, and may affect activity content relevant to products or services of an **ineligible company**, defined as an entity whose primary business is producing, marketing, selling, re-selling, or distributing healthcare products used by or on patients.

Research grants from **ineligible companies** are financial relationships that should be disclosed, even if the funds go to the researcher's institution and not to the individual researcher.

The author's relationships/activities/interests should be defined broadly and not only related to the manuscript in question. For example, if your manuscript pertains to the epidemiology of shock, you should declare all relationships with manufacturers of treatments used in shock, even if that form of treatment is not mentioned in the manuscript.

According to federal regulations approved by the US Senate, any amount equal to above $10 USD must be disclosed. Although disclosure of the total amount is not required on this form. Authors are encouraged to search the CMS Open Payments Database found at https://openpaymentsdata.cms.gov and report on the JTACS Conflict of Interest Disclosure form ALL COI, and any other conflicts related or unrelated to the manuscript being submitted to the Journal for the last 36 months/3 years.

In item #1 below, report all support for the work reported in this manuscript without time limit. For all other items, the time frame for disclosure is the past 36 months.

If the article is accepted, all author JTACS COI forms will be published as supplemental material with the article.

		Name all entities with whom you have this relationship or indicate none (add rows as needed)	Specifications/Comments (e.g., if payments were made to you or to your institution)
**Time frame: Since the initial planning of the work**			
**1**	All support for the present manuscript (e.g., funding, provision of study materials,	☒ **None**	
			Click the tab key to add additional rows.

		Name all entities with whom you have this relationship or indicate none (add rows as needed)	Specifications/Comments (e.g., if payments were made to you or to your institution)
	medical writing, article processing charges, etc.) **No time limit for this item.**		
	**Time frame: past 36 months**		
**2**	Grants or contracts from any entity (if not indicated in item #1 above).	☒ **None**	
**3**	Royalties or licenses	☒ **None**	
**4**	Consulting fees	☒ **None**	
**5**	Payment or honoraria for lectures, presentations, speakers bureaus, manuscript writing or educational events	☒ **None**	
**6**	Payment for expert testimony	☒ **None**	
**7**	Support for attending meetings and/or travel	☒ **None**	

		Name all entities with whom you have this relationship or indicate none (add rows as needed)	Specifications/Comments (e.g., if payments were made to you or to your institution)
8	Patents planned, issued or pending	☒ None	
9	Participation on a Data Safety Monitoring Board or Advisory Board	☒ None	
10	Leadership or fiduciary role in other board, society, committee or advocacy group, paid or unpaid	☒ None	
11	Stock or stock options	☒ None	
12	Receipt of equipment, materials, drugs, medical writing, gifts or other services	☒ None	
13	Other financial or non-financial interests	☒ None	
14	Family Disclosure. Disclose any financial associations involving a spouse, partner, or children	☒ None	

Please place an "X" next to the following statement to indicate your agreement: [illegible] 09.12.2025

☒ I certify that I have answered every question and have not altered the wording of any of the questions on this form.
